# Sugar-sweetened beverage intake and chronic low back pain

**DOI:** 10.3389/fnut.2024.1418393

**Published:** 2024-07-03

**Authors:** Yanting Wang, Yuchen Tang, Zhichun Li, Changhai Jiang, Wei Jiang, Zhenming Hu

**Affiliations:** ^1^Department of Orthopedics, Orthopedic Laboratory of Chongqing Medical University, The First Affiliated Hospital of Chongqing Medical University, Chongqing, China; ^2^Department of Orthopedics, The People’s Hospital of Chuxiong Yi Autonomous Prefecture, Chuxiong, Yunnan, China; ^3^Department of General Surgery, Caoxian People's Hospital, Heze, Shandong, China; ^4^Department of Orthopedics, University-Town Hospital of Chongqing Medical University, Chongqing, China

**Keywords:** sugar-sweetened beverage, chronic low back pain, NHANES, smoking, hypertension

## Abstract

**Background:**

The consumption of sugar-sweetened beverages (SSBs) has become a major public health problem globally. However, no studies have specifically examined the relationship between SSB intake and chronic low back pain (CLBP). Therefore, the present study aimed to investigate the relationship between SSB intake and the risk of CLBP.

**Methods:**

This cross-sectional study enrolled participants aged 20 to 69 from the National Health and Nutrition Examination Survey. CLBP was defined as persistent LBP for a consecutive three-month period. Furthermore, SSB intake was assessed and calculated based on dietary recall interviews. Moreover, survey-weighted logistic regression models were employed to evaluate the association between SSB intake and the risk of CLBP, while the restricted cubic spline (RCS) analysis was used to determine whether there were nonlinear associations between SSB intake and CLBP risk. In addition, subgroup analysis was performed using stratification and interaction analysis for all covariates.

**Results:**

A total of 4,146 participants (mean age: 43.405 years) were enrolled in the final analysis. The results of survey-weighted logistic regression models showed that SSB consumption was significantly associated with an increased risk of CLBP among individuals aged 20 to 69 years. Moreover, the results of subgroup analysis and interaction analysis demonstrated that the association between SSB intake and the risk of CLBP was modified by smoking status and hypertension. Specifically, the SSB intake-associated CLBP risk was more pronounced among current smokers or individuals with hypertension.

**Conclusion:**

Reduction of SSB consumption might contribute to the prevention of CLBP for individuals aged 20 to 69 years. Moreover, current smokers or individuals with hypertension should be more vigilant about the SSB intake-associated CLBP risk. Nevertheless, caution should be exercised when interpreting the results of this study, as further research is necessary to explore the association between SSB consumption and CLBP, given the limitations of the current study.

## Introduction

Low back pain (LBP) is a prevalent musculoskeletal disorder affecting a significant proportion of adults globally, with a prevalence ranging from 50 to 80% (1, 2). Chronic LBP (CLBP), characterized by pain persisting for more than 3 months and strongly associated with intervertebral disc degeneration (3, 4), is recognized as a major contributor to disability globally (5, 6), and this issue is exacerbated by the aging population and the growth of the population worldwide (7). Currently, there is a growing emphasis on the early prevention of CLBP due to the lack of effective therapeutic strategies. Moreover, cumulative evidence indicates that the pathogenesis of CLBP is complex and is associated with several risk factors, such as age, lifestyle factors, and dietary choices (8, 9). In addition, substantial evidence has implicated that diet and lifestyle interventions have beneficial effects on reducing the risk and improving the condition of CLBP (10, 11). Therefore, the exploration of risk factors for CLBP from the diet and lifestyle perspective has gained considerable attention in recent years and may provide theoretical guidance in the early prevention of CLBP.

Sugar-sweetened beverages (SSBs), including carbonated soft drinks, fruit drinks, and energy drinks, has been demonstrated to be leading sources of added sugars in the diet and to be associated with several adverse health outcomes, such as obesity, oral health, diabetes, and cardiovascular diseases (12–16). Therefore, the consumption of SSBs remains a major public health problem globally (17, 18), which also results in the formulation and implementation of interventions and policies, such as sugary drink warnings or SSB tax (19, 20). Previous evidence has suggested a potential link between high SSB consumption and musculoskeletal disorders, such as low bone mineral density and gout (21, 22). However, to the best of our knowledge, no studies have specifically examined the relationship between SSB intake and CLBP. In addition, it remains unknown whether there are potential factors that modify the association between SSB consumption and the risk of CLBP. Therefore, it is necessary to investigate and understand the relationship between SSB intake and CLBP further, which is crucial and may provide valuable insights into the role of dietary factors in the development and management of CLBP.

Based on the background above, the present study aimed to investigate the relationship between SSB intake and the risk of CLBP and to explore the potential factors that modified the relationship between SSB intake and CLBP, which may have important implications for public health policies, prevention strategies, and patient education regarding CLBP and SSB consumption.

## Materials and methods

### Study design and population

This cross-sectional study included participants from the National Health and Nutrition Examination Surveys (NHANES) 2009–2010, in which the data utilized in the present study is openly accessible on the NHANES website.^1^ Participants who received the Inflammatory Arthritis Questionnaire, which was employed for CLBP assessment, were included in the present study. Moreover, the exclusion criteria for participants were listed as follows: (i) with missing data on SSB intake; (ii) with missing data on covariates. Furthermore, ethical approval for the NHANES was obtained from the ethics review board of the National Center for Health Statistics (23). All participants in the NHANES study were duly provided with and acknowledged informed consent (24). The present study conducted was a secondary analysis of deidentified, publicly available data, thus obviating the need for ethics approval. Additional comprehensive information was accessible on the NHANES website (25).

### CLBP assessment

CLBP, in which the definition was employed with reference to several previous studies (26, 27), was evaluated using the Inflammatory Arthritis Questionnaire [offering interview data pertaining to chronic back pain, Inflammatory Back Pain (IBP), and Spondyloarthritis (Spondyloarthritis or Spinal Arthritis)] (28, 29), with the study population consisting of a representative sample of United States adults aged 20 to 69 years. Moreover, all participants who received the Inflammatory Arthritis Questionnaire underwent the same assessments for CLBP, and a participant who was asked the question, “Had low back pain 3 months in a row?” met the criteria for CLBP if they reported experiencing persistent LBP for a consecutive three-month period. Detailed information on the Inflammatory Arthritis Questionnaire is available on the NHANES website (28, 29).

### SSB intake

SSB intake was evaluated through 24-h dietary recall interviews, which captured the consumption of various foods and beverages in the preceding 24 h. All reported food and beverage items were meticulously coded using the US Department of Agriculture (USDA) Food and Nutrient Database. Soft drinks, fruit drinks (not 100%), sports drinks, energy drinks, nutritional beverages, smoothies, grain drinks, carbonated water, and sweetened coffee and tea were considered the SSBs in the present study. The caloric content and nutrient composition of SSBs were determined by analyzing the reported quantities of food and beverages in conjunction with the nutrient data provided by the National Center for Health Statistics. Additional information regarding the methodology of dietary recall interviews can be accessed on the NHANES website (30).

### Covariates

Several demographic variables and variables considered as potential confounders of the relationship between SSB intakes and the risk of CLBP were included as the covariates in the subsequent analysis. Age, sex, race/ethnicity, education levels, body mass index (BMI), smoking status, drinking status, physical activity levels (mins/week, which were assessed by the Global physical activity questionnaire (GPAQ) (31) and included five aspects: vigorous work-related activity, moderate work-related activity, walking or bicycling for transportation, vigorous leisure-time physical activity, and moderate work-related activity), hypertension (diagnosed by doctors), diabetes (diagnosed by doctors), cancer (diagnosed by doctors), C-reactive protein (CRP), and total energy intake were selected as the covariates of the present study.

### Statistical analysis

Baseline characteristics of the study population were reported as means [standard errors (SEs)] for continuous variables and unweighted numbers (weighted proportions) for categorical variables, in which nationally representative estimates were calculated for all analyses by utilizing the recommended NHANES examinations sample weights (32). Furthermore, the differences between individuals with and without CLBP were assessed by survey-weighted linear regression models for continuous variables and survey-weighted Chi-square test for categorical variables. Moreover, the weighted binomial logistic regression models were employed to determine the association between SSB intake and the risk of CLBP and to calculate the odds ratios (ORs) and 95% confidence intervals (CIs), while restricted cubic spline (RCS) curves based on survey-weighted binomial logistic regression models were used to examine whether there were significant nonlinear associations between SSB intake and the risk of CLBP. In addition, subgroup analysis was performed using stratification and interaction analysis for all covariates mentioned above to determine whether there were potential factors that modified the association between SSB intake and the risk of CLBP. Statistical analyses were performed using R software version 4.2.1^2^ and EmpowerStats version 4.2.^3^ Two-sided *p*-values were utilized, with significance defined as *p* < 0.05.

## Results

### Study population selection

**Figure 1:**
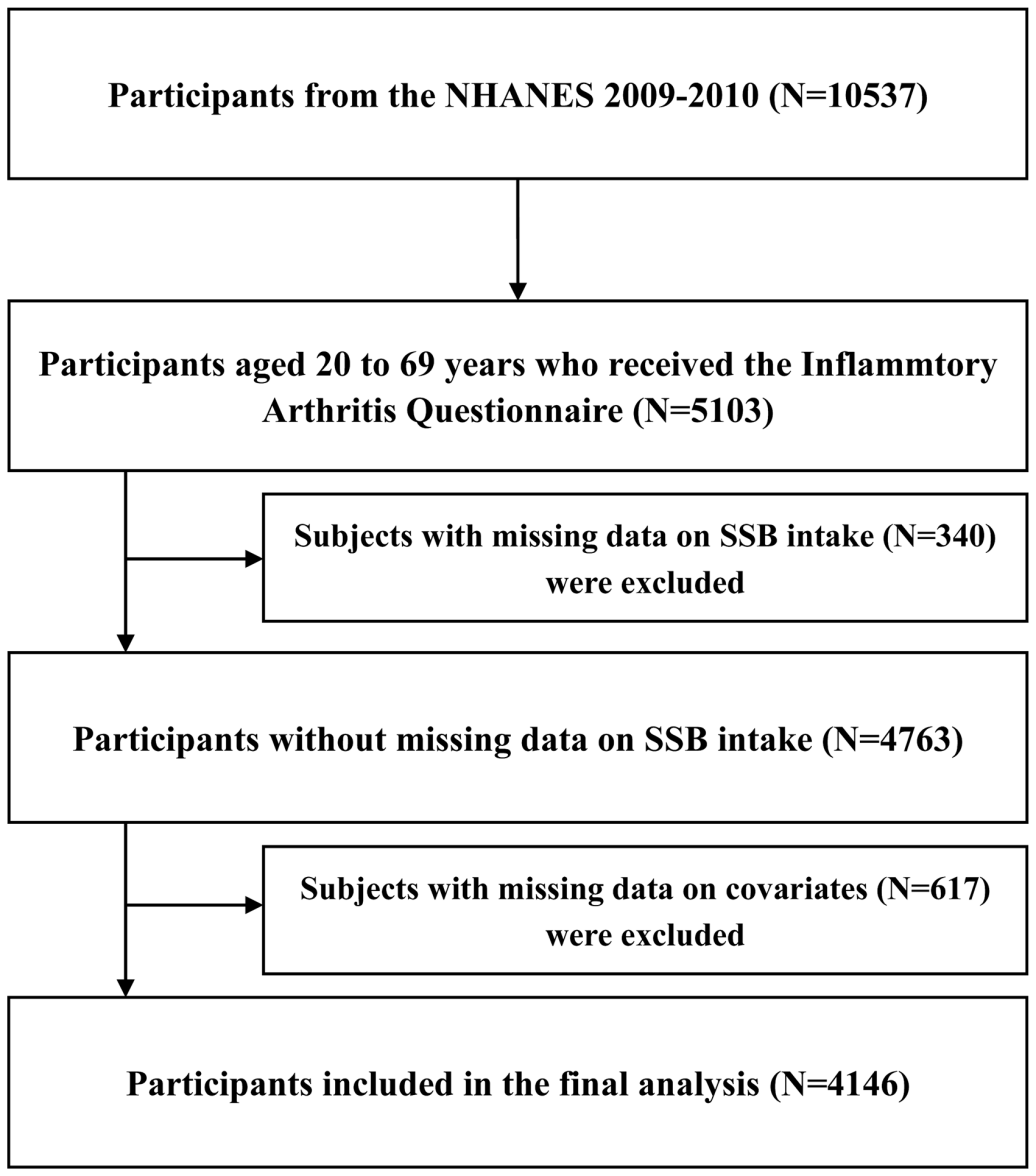
Flowchart of participants selection. CLBP, chronic low back pain; NHANES, National Health and Nutrition Examination Survey; SSB, sugar-sweetened beverage.

Overall, 10,537 participants from the NHANES 2009–2010 were included in this cross-sectional study, in which 5,103 participants aged 20–69 years received the Inflammatory Arthritis Questionnaire. Furthermore, individuals with incomplete data regarding SSB consumption (*N* = 340) or covariates (*N* = 617) were excluded from the analysis. Ultimately, a cohort of 4,146 participants was deemed suitable for inclusion in the final analysis. The selection process of the study population is visually represented in Figure 1.

### Baseline characteristics

Finally, 4,146 participants aged 20 to 69 years were included in the final analysis, and weighted samples of participants represent a population of 171,120,866. The mean age of the study population was 43.405 (0.382) years, and 50.062% were women. Furthermore, participants with CLBP tended to be older and show a higher prevalence of obesity, smoking, hypertension, diabetes, and cancer than those without CLBP. Moreover, the mean SSB intakes of the overall population, participants with CLBP, and those without CLBP were 120.017 (5.452) kcal/d, 149.249 (9.885) kcal/d, 114.911 (5.589) kcal/d, respectively, in which participants with CLBP showed significantly higher SSB intakes than those without CLBP. Other baseline characteristics of the study population are listed in Table 1.

**Table 1:** Baseline characteristics.

Characteristic	Total (*N* = 4,146)^a^	Without CLBP (*N* = 3,549)^a^	With CLBP (*N* = 597)^a^	*p*-value
*Age (years)*	43.405 (0.382)	42.963 (0.375)	45.939 (0.765)	< 0.001
*Sex*				0.644
Men	2,048 (49.938)	1,771 (50.146)	277 (48.745)	
Women	2,098 (50.062)	1,778 (49.854)	320 (51.255)	
*Race/ethnicity*				0.070
Non-Hispanic White	1,876 (68.345)	1,551 (67.350)	325 (74.043)	
Non-Hispanic Black	742 (11.218)	651 (11.491)	91 (9.656)	
Mexican American	847 (8.847)	745 (8.992)	102 (8.018)	
Other races	681 (11.590)	602 (12.168)	79 (8.283)	
*Education level*				0.027
Under high school	1,106 (17.044)	937 (16.605)	169 (19.557)	
High school or equivalent	944 (22.000)	793 (21.277)	151 (26.142)	
Above high school	2,096 (60.956)	1,819 (62.118)	277 (54.301)	
BM^b^				0.013
Normal	1,148 (30.308)	1,023 (31.723)	125 (22.206)	
Overweight	1,375 (32.591)	1,185 (32.399)	190 (33.693)	
Obese	1,623 (37.101)	1,341 (35.878)	282 (44.101)	
*Smoking status*				< 0.001
Never	2,264 (55.392)	2035 (57.570)	229 (42.921)	
Former	868 (22.781)	709 (21.933)	159 (27.635)	
Current	1,014 (21.828)	805 (20.497)	209 (29.444)	
Drinking status^c^				0.406
Never	445 (8.571)	393 (8.691)	52 (7.879)	
Former	537 (10.224)	464 (10.012)	73 (11.440)	
Current	3,164 (81.205)	2,692 (81.297)	472 (80.682)	
*PA levels (mins/week)*	688.890 (35.566)	675.499 (34.302)	765.556 (65.871)	0.115
*Hypertension*				< 0.001
Yes	1,171 (25.093)	921 (23.304)	250 (35.339)	
No	2,975 (74.907)	2,628 (76.696)	347 (64.661)	
*Diabetes*				0.009
Yes	400 (6.713)	313 (6.064)	87 (10.430)	
No	3,746 (93.287)	3,236 (93.936)	510 (89.570)	
*Cancer*				0.005
Yes	258 (7.440)	199 (6.760)	59 (11.329)	
No	3,888 (92.560)	3,350 (93.240)	538 (88.671)	
*CRP (mg/dL)*	0.360 (0.017)	0.352 (0.017)	0.408 (0.032)	0.055
*Total energy intake (kcal/d)*	2198.543 (19.625)	2193.157 (19.816)	2229.378 (58.626)	0.557
*SSB intake (kcal/d)*	120.017 (5.452)	114.911 (5.589)	149.249 (9.885)	0.002

a Unweighted number.

b Normal: <25 kg/m^2^; Overweight: <30 but ≥ 25 kg/m^2^; Obese: ≥30 kg/m^2^.

c Never: participants who did not have at least 12 alcohol drinks in a lifetime; Former: participants who had at least 12 alcohol drinks in a lifetime but did not have at least 12 alcohol drinks for last 1 year; Current: participants who had at least 12 alcohol drinks in a lifetime and had at least 12 alcohol drinks for last 1 year.

BMI, body mass index; CLBP, chronic low back pain; CRP, C-reactive protein; PA, physical activity; SSB, sugar-sweetened beverage.

### Association between SSB intake and CLBP

**Figure 2:**
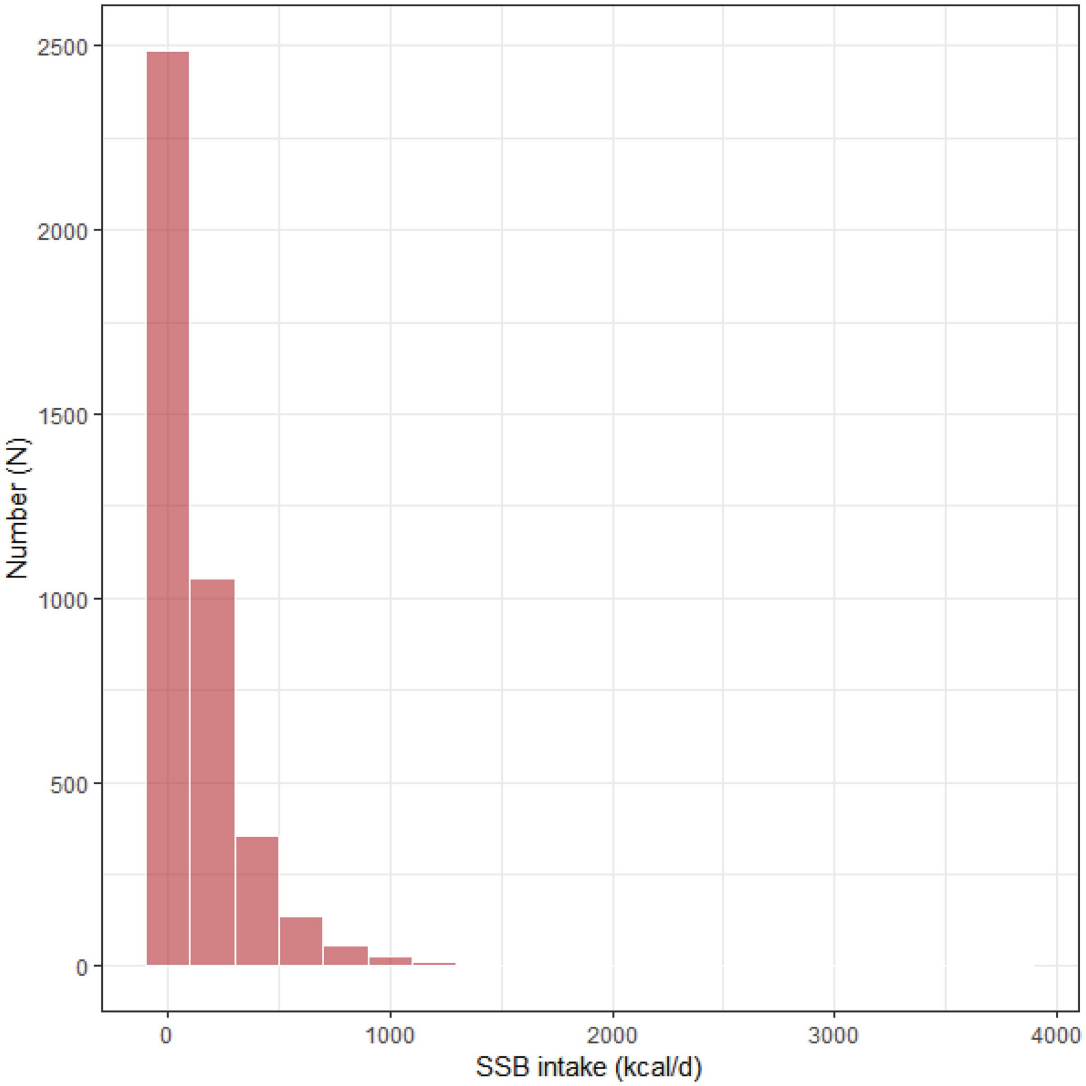
The data distribution of SSB intake. SSB, sugar-sweetened beverage.

**Figure 3:**
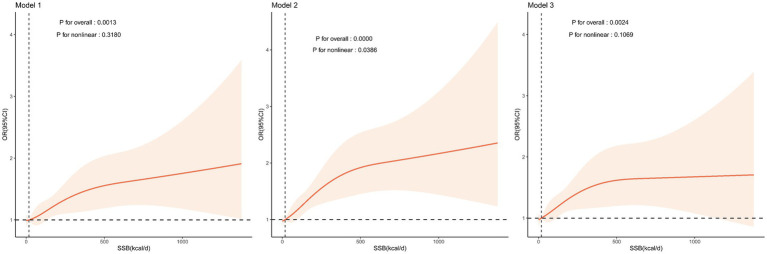
Relationship between SSB intake and the risk of CLBP. Model 1: adjustment for no covariates; Model 2: adjustment for age, sex, and race/ethnicity; Model 3: adjustment for all covariates. Data were fitted by a restricted cubic spline linear regression model, and the model was conducted with 4 knots at the 5th, 35th, 65th, 95th percentiles of SSB intake (reference is the median). Solid lines indicate OR values, and shadow shape indicates 95% CIs. CLBP, chronic low back pain; CI, confidence interval; OR, odds ratio; SSB, sugar-sweetened beverage.

The results of weighted logistic regression models (Table 2) indicated that higher SSB intake (as a continuous variable) was associated with an increased risk of CLBP with or without adjustment for covariates. Moreover, when SSB intake was converted to a categorical variable (no SSB intake: 0 kcal/d, low SSB intake: 0–199 kcal/d, and high SSB intake: ≥200 kcal/d) according to the data distribution of SSB intake (Figure 2), participants with high SSB intake showed an elevated risk of CLBP compared with those with no SSB intake with or without adjusting for covariates. In addition, the results of RCS models (Figure 3) suggested that there were no significant nonlinear associations between SSB intake and the risk of CLBP with or without adjustment for covariates (P for nonlinear >0.05).

**Table 2:** Association between SSB intake and the risk of CLBP.

	Model 1^a^		Model 2^b^		Model 3^c^	
	OR (95%CI)	*p*-value	OR (95%CI)	*p*-value	OR (95%CI)	*p*-value
SSB intake (continuous variable) (Per 100 kcal/d increase)	1.071 (1.035, 1.107)	<0.001	1.101 (1.059, 1.144)	<0.001	1.069 (1.022, 1.117)	0.006
SSB intake (categorical variable)						
Group 1: 0 kcal/d	Ref (1)	–	Ref (1)	–	Ref (1)	–
Group 2: 1–199 kcal/d	1.216 (0.763, 1.938)	0.383	1.237 (0.766, 1.998)	0.342	1.163 (0.716, 1.889)	0.519
Group 3: ≥200 kcal/d	1.653 (1.232, 2.217)	0.003	1.939 (1.432, 2.624)	<0.001	1.647 (1.163, 2.333)	0.008
P for trend	0.005		0.002		0.018	

a Adjustment for no covariates were adjusted.

b Adjustment for age, sex, and race/ethnicity were adjusted.

c Adjustment for all covariates (including age, sex, race/ethnicity, education levels, BMI, smoking status, drinking status, PA levels, hypertension, diabetes, cancer, CRP, and total energy intake) were adjusted.

BMI, body mass index; CI, confidence interval; CLBP, chronic low back pain; CRP, C-reactive protein; OR, odds ratio; PA, physical activity; SSB, sugar-sweetened beverage.

### Subgroup analysis

**Figure 4:**
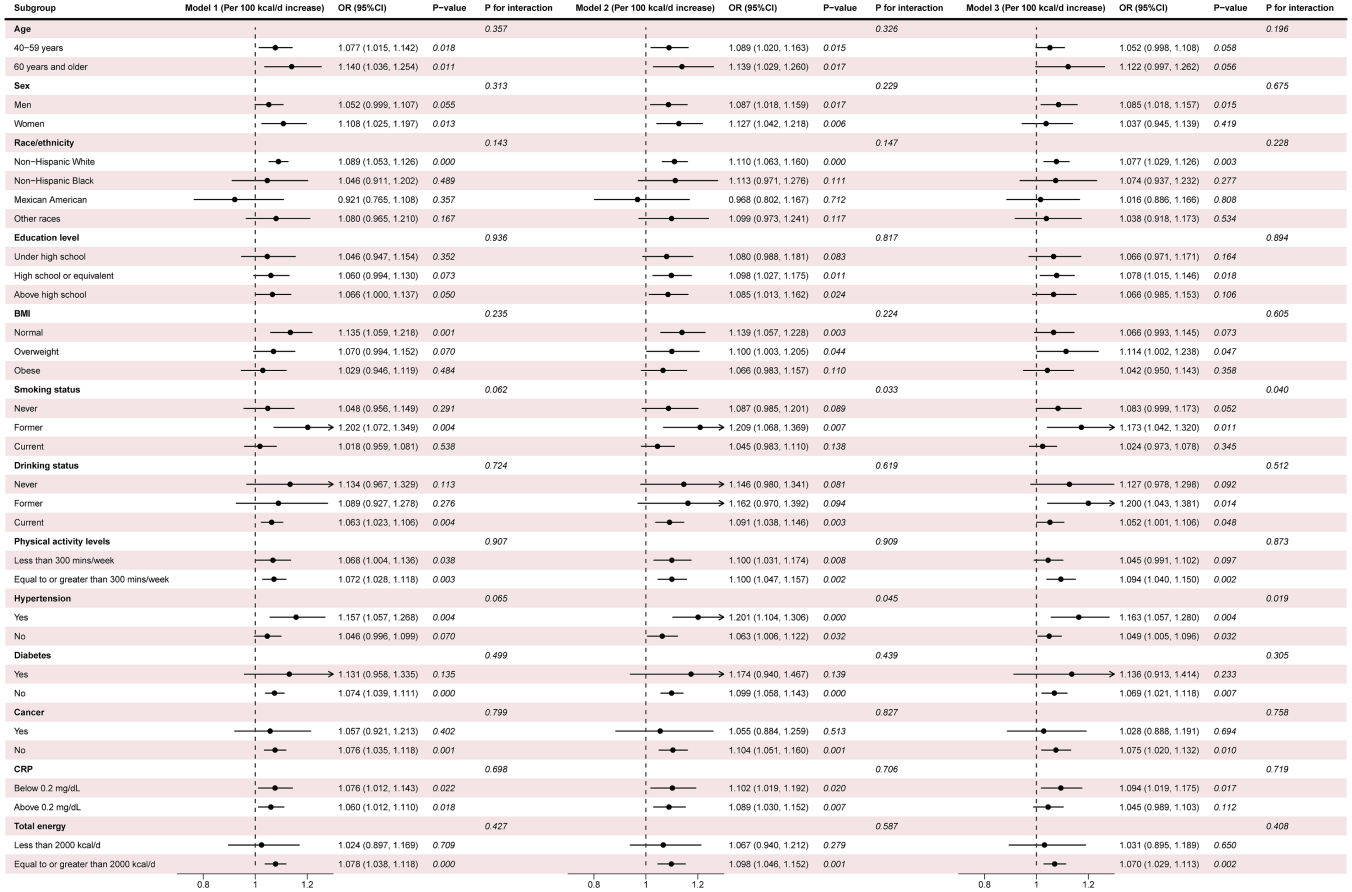
Subgroup analysis and interaction testing. Model 1: adjustment for no covariates; Model 2: adjustment for age, sex, and race/ethnicity; Model 3: adjustment for all covariates. Covariates were not adjusted when stratified by their respective variables. 25(OH) D, 25-hydroxyvitamin D; BMD, bone mineral density; BMI, body mass index; CI, confidence interval; CRP, C-reactive protein; OR, odds ratio.

The results of subgroup analysis (Figure 4) demonstrated that higher SSB intake was associated with an increased risk of CLBP, which was observed in most of the subgroups with or without adjusting for covariates. Moreover, the results of interaction analysis suggested (Figure 4) that the association between SSB intake and the risk of CLBP were modified by smoking status and hypertension after adjusting for covariates (P for interaction <0.05). Furthermore, the results of weighted logistic regression analysis (Table 3) showed that current smokers, irrespective of the SSB intake, showed a significantly elevated risk of CLBP, and former smokers with high SSB intake showed a significantly increased risk of CLBP compared with never smokers with no SSB intake with or without adjustment for covariates. In addition, this study observed (Table 4) that only the hypertension group with high SSB intake showed a significantly elevated risk of CLBP compared with the non-hypertension group with no SSB intake after adjusting for all covariates.

**Table 3:** Association between SSB intake, smoking status, and the risk of CLBP.

SSB intake	Smoking status	Model 1^a^		Model 2^b^		Model 3^c^	
		OR (95%CI)	*p*-value	OR (95%CI)	*p*-value	OR (95%CI)	*p*-value
No SSB intake: 0 kcal/d	Never	Ref (1)	–	Ref (1)	–	Ref (1)	–
	Former	1.671 (0.970, 2.879)	0.061	1.479 (0.695, 3.144)	0.198	1.511 (0.911, 2.506)	0.103
	Current	2.211 (1.396, 3.501)	0.004	2.251 (1.228, 4.126)	0.024	2.252 (1.383, 3.666)	0.003
Low SSB intake: 1–199 kcal/d	Never	1.264 (0.639, 2.501)	0.451	1.264 (0.497, 3.212)	0.483	1.202 (0.619, 2.337)	0.565
	Former	1.962 (0.901, 4.270)	0.081	1.750 (0.587, 5.220)	0.201	1.585 (0.701, 3.588)	0.249
	Current	2.482 (1.329, 4.636)	0.010	2.632 (1.107, 6.260)	0.038	2.668 (1.474, 4.830)	0.003
High SSB intake: ≥200 kcal/d	Never	1.700 (0.991, 2.917)	0.053	1.977 (0.940, 4.160)	0.062	1.835 (1.121, 3.005)	0.019
	Former	3.696 (2.026, 6.741)	0.001	3.759 (1.774, 7.968)	0.011	3.372 (1.912, 5.949)	<0.001
	Current	2.380 (1.302, 4.352)	0.011	2.792 (1.299, 6.001)	0.024	2.618 (1.456, 4.705)	0.003

a Adjustment for no covariates.

b Adjustment for age, sex, and race/ethnicity.

c Adjustment for all covariates (including age, sex, race/ethnicity, education levels, BMI, drinking status, PA levels, hypertension, diabetes, cancer, CRP, and total energy intake).

BMI, body mass index; CI, confidence interval; CLBP, chronic low back pain; CRP, C-reactive protein; OR, odds ratio; PA, physical activity; SSB, sugar-sweetened beverage.

**Table 4:** Association between SSB intake, hypertension, and the risk of CLBP.

SSB intake	History of hypertension	Model 1^a^		Model 2^b^		Model 3^c^	
		OR (95%CI)	*p*-value	OR (95%CI)	*p*-value	OR (95%CI)	*p*-value
No SSB intake: 0 kcal/d	No	Ref (1)	–	Ref (1)	–	Ref (1)	–
	Yes	1.806 (1.311, 2.489)	0.002	1.604 (1.120, 2.298)	0.018	1.333 (0.974, 1.824)	0.070
Low SSB intake: 1–199 kcal/d	No	1.337 (0.719, 2.486)	0.326	1.364 (0.702, 2.650)	0.296	1.307 (0.706, 2.420)	0.371
	Yes	1.711 (0.961, 3.048)	0.065	1.519 (0.807, 2.861)	0.157	1.236 (0.653, 2.338)	0.492
High SSB intake: ≥200 kcal/d	No	1.432 (0.948, 2.163)	0.082	1.608 (0.980, 2.639)	0.057	1.347 (0.877, 2.068)	0.160
	Yes	4.298 (2.522, 7.324)	<0.0001	4.304 (2.356, 7.862)	0.001	3.411 (1.753, 6.637)	0.001

a Adjustment for no covariates.

b Adjustment for age, sex, and race/ethnicity.

c Adjustment for all covariates (including age, sex, race/ethnicity, education levels, BMI, smoking status, drinking status, PA levels, diabetes, cancer, CRP, and total energy intake).

BMI, body mass index; CI, confidence interval; CLBP, chronic low back pain; CRP, C-reactive protein; OR, odds ratio; PA, physical activity; SSB, sugar-sweetened beverage.

## Discussion

Overall, this cross-sectional study observed that SSB consumption was significantly associated with an increased risk of CLBP among individuals aged 20 to 69 years. Moreover, we found that the association between SSB intake and the risk of CLBP was modified by smoking status and hypertension, in which the SSB intake-associated CLBP risk was more pronounced among current smokers or individuals with hypertension.

SSB consumption, which has been demonstrated to be associated with several adverse health outcomes (12–16), has become a major public health problem worldwide (17, 18). In the present study, we observed a significant association between the consumption of SSBs and an increased CLBP risk, the specific mechanisms of which are yet to be elucidated. However, we speculate that there are several possible causes of this phenomenon, including inflammatory, metabolic, nutritional, lifestyle, and psychological factors. For example, SSBs are known to have high levels of added sugars, which can lead to elevated inflammation levels in the body (33), which is believed to play a role in the development and persistence of pain, including CLBP (34–36). Furthermore, regular consumption of SSBs has been demonstrated to be associated with an elevated risk of weight gain, obesity, or diabetes (15, 37), which are also considered important risk factors for CLBP reported by numerous studies (8, 26, 38). Moreover, it is possible that individuals who consume higher amounts of SSBs might also have additional risk factors for CLBP, such as a sedentary lifestyle and higher stress levels (39, 40), which may be a possible explanation for the association between SSB consumption and an increased risk of CLBP. In addition, it should be noted that simple carbohydrates, such as fructose, have been demonstrated to have a direct nociceptive effect on pain sensation (41), which is also a probable cause for the association between high SSB consumption and the increased risk of CLBP. However, additional investigations are required to support our speculation due to the cross-sectional study design, which does not allow causal associations to be drawn.

Interestingly, this study observed that the association between SSB intake and the risk of CLBP was modified by smoking status and hypertension, in which the SSB intake-associated CLBP risk was more pronounced among current smokers or individuals with hypertension, suggesting that there might be a synergistic effect between SSB intake and smoking, as well as hypertension, in CLBP. On the one hand, both smoking and hypertension can contribute to elevated inflammation levels in the body (42, 43). SSBs, with their high sugar content, may further exacerbate inflammation levels (33). The synergistic effects of smoking, hypertension, and SSB consumption may lead to an even higher level of systemic inflammation, which has been demonstrated to be associated with an increased risk of CLBP (34–36). On the other hand, current smokers or individuals with hypertension may have other lifestyle factors that contribute to their increased risk of CLBP when combined with SSB consumption, such as poor dietary habits or a sedentary lifestyle, all of which can independently contribute to the development of CLBP (39, 40, 44). However, it should be noted that these potential reasons mentioned above are based on observations and correlations, and further research is needed to fully understand the underlying mechanisms and causality between SSB intake, smoking, hypertension, and CLBP.

The main findings of this study have implications for future clinical practice. To the best of our knowledge, this study is the first to investigate the association between SSB consumption and CLBP risk. Moreover, this study found a significant association between the consumption of SSBs and an increased risk of CLBP among individuals aged 20 to 69 years, which implies that SSB consumption may contribute to the development or progression of CLBP, while the reduction in SSB intake may serve to protect from CLBP. Furthermore, this study observed that SSB intake-associated CLBP risk was more pronounced among current smokers or individuals with hypertension. Therefore, these special populations need to be aware of the potential synergistic impact on CLBP risk. In addition, limiting SSB intake and addressing other risk factors, such as smoking and hypertension, may help reduce the burden of CLBP in the population.

This study is subject to certain limitations. Firstly, the cross-sectional study design utilized in this research precludes the establishment of a causal relationship between SSB intake and the risk of CLBP. Secondly, data on SSB intake and various covariates, including smoking status and history of hypertension, were obtained through dietary recall interviews or self-report questionnaires, potentially introducing reporting bias or recall bias. Thirdly, it should be noted that the participants in this study were drawn from the NHANES database, which represents the US population, suggesting the generalizability of the findings to populations in other countries or regions may be limited. Fourthly, the sample size of participants with CLBP was relatively small, which might influence the precision of estimation. Consequently, further research investigating the association between SSB consumption and the risk of CLBP is warranted to enhance the robustness of the evidence.

## Conclusion

SSB consumption was significantly associated with an elevated risk of CLBP among individuals aged 20 to 69 years, suggesting that the reduction in SSB intake might contribute to the prevention of CLBP. Moreover, the association between SSB intake and CLBP risk was modified by several lifestyles and diseases, including smoking and hypertension, suggesting such individuals should be more vigilant about the SSB intake-associated CLBP risk. However, the results from this study should be interpreted with caution, and additional studies are required in the future further to investigate the relationship between SSB consumption and CLBP, considering that there are several limitations of the present study.

## Data availability statement

Publicly available datasets were analyzed in this study. This data can be found here: the datasets obtained and analyzed in this study are publicly available on the NHANES database (https://www.cdc.gov/nchs/nhanes/index.htm).

## Ethics statement

The NHANES was granted approval by the National Center for Health Statistics Ethics Review Board. All participants from the NHANES were duly provided with and acknowledged the informed consent. The studies were conducted in accordance with the local legislation and institutional requirements. The participants provided their written informed consent to participate in this study.

## Author contributions

YW: Conceptualization, Data curation, Formal analysis, Methodology, Writing – original draft, Writing – review & editing. YT: Formal analysis, Investigation, Methodology, Validation, Writing – original draft, Writing – review & editing. ZL: Investigation, Validation, Writing – review & editing. CJ: Validation, Writing – review & editing. WJ: Conceptualization, Funding acquisition, Supervision, Writing – review & editing. ZH: Conceptualization, Methodology, Supervision, Writing – review & editing.

## Footnotes

^1^ https://www.cdc.gov/nchs/nhanes/index.htm

^2^ https://cran.r-project.org/

^3^ http://www.empowerstats.com
